# Association of CIDEB gene promoter methylation with overweight or obesity in adults

**DOI:** 10.18632/aging.204032

**Published:** 2022-04-27

**Authors:** Zhiguang Ping, Zhaoyan Guo, Ming Lu, Yanzi Chen, Li Liu

**Affiliations:** 1 College of Public Health, Zhengzhou University, Zhengzhou, Henan, China; 2 Nursing Department of Jiaozuo People’s Hospital, Jiaozuo, Henan, China; 3 Henan Huapu Pharmaceutical Technology Co., Ltd., Zhengzhou, Henan, China; 4 School of Basic Medical Sciences, Zhengzhou University, Zhengzhou, Henan, China

**Keywords:** CIDEB, DNA methylation, obesity, adipose tissue, haplotype

## Abstract

Objective: To explore the association of the methylation level of cell death-inducing DFF45-like effector B (CIDEB) gene promoter with overweight or obesity in the abdominal subcutaneous adipose tissue (SAT) and omental adipose tissue (OAT) of adults.

Methods: A total of 61 patients undergoing abdominal surgery in the hospital were selected with an average age of 51.87 years. According to the diagnostic criteria of Chinese adult obesity, the subjects were divided into normal-weight group (n = 28) and overweight/obesity group (n = 33). CIDEB promoter methylation level in abdominal SAT and OAT was detected by the MethylTarget technology, then its relationship with overweight or obesity was analyzed.

Results: (1) There were no statistical differences between the normal-weight group and overweight/obesity group in Methylation levels of 16 CpG sites in the CIDEB gene promoter sequence. (2) The methylation level of OAT was higher than that of SAT, and there were significant differences in 16 CpG sites. (3) There were 3 statistically significant haplotypes between the normal-weight group and overweight/obesity group (2 in SAT and 1 in OAT).

Conclusions: The methylation level of CIDEB gene promoter in abdominal SAT and OAT may be related to overweight or obesity in adults, and the specific regulatory mechanism needs to be further studied.

## INTRODUCTION

Obesity is a chronic metabolic disease caused by a variety of factors, characterized by disorders of body energy metabolism and excessive fat accumulation. It is closely related to the occurrence of chronic diseases such as type 2 diabetes, hypertension, cardiovascular disease and tumor, and has become a major risk factor threatening human health [1, 2]. The latest data from the World Health Organization (WHO) in 2016 show that more than 1.9 billion adults worldwide are overweight, of which more than 650 million are obese [3]. Obesity has become widespread worldwide and is currently one of the health crises in the world.

Obesity is the result of the interaction of genetic, environmental, and behavioral factors. Family and twin studies have shown that heritability estimates of 40-70% [4], which allows modern molecular genetics to deeply explore the mechanism of the occurrence and development of obesity. DNA methylation is one of the most deeply studied epigenetic modifications, which is highly related to adipocyte differentiation and the occurrence of obesity [5, 6]. It affects the growth and development of adipose tissue by regulating the expression of adipocyte differentiation transcription factors, transcription cofactors, and other adipocytes metabolism-related genes [7].

Cell death-including DFF45-like effector B (CIDEB), located on human chromosome 14q11, can affect gene expression in multiple metabolic pathways and signaling networks, such as lipid droplet formation, lipogenesis, glycolysis, and gluconeogenesis and so on [8–10]. As an important lipid droplet surface protein, CIDEB promotes the lipidation and maturation of very low density lipoprotein (VLDL) by binding to apolipoprotein B and then regulates fat metabolism [11]. The study found that CIDEB-null mice displayed significantly increased body metabolism, which could resist obesity and liver fat degeneration induced by high-fat diet [12]. Compared with normal wild-type mice, CIDEB-null mice have smaller lipid droplets, lower proportion of white adipose tissue (WAT), and significantly lower plasma triglyceride (TG) and fatty acid contents [12, 13]. Therefore, the CIDEB gene plays an important role in maintaining lipid homeostasis and energy metabolism of the whole organism [14].

At present, related studies on CIDEB and lipid metabolism are mostly focused on gene knockout, and most of them are animal experiments on mice. The study on the methylation level of CIDEB gene promoter in obesity has not been reported. The purpose of this study is to explore the relationship between CIDEB and overweight/obesity by comparing the CIDEB gene promoter methylation level in abdominal subcutaneous adipose tissue (SAT) and omental adipose tissue (OAT) between normal-weight and overweight/obesity groups, as to provide a theoretical basis for gene-targeted therapy of obesity.

## MATERIALS AND METHODS

### Materials

### Subjects

According to the inclusion and exclusion criteria, 61 patients with abdominal surgery from a municipal hospital of Henan province in China were selected, with an average age of 51.87±14 years, including 10 males and 51 females. The case data of the subjects were collected, including the patient's height, weight, waist circumference (WC), hip circumference, blood pressure, fasting plasma glucose (FPG), blood lipids, and other indicators. According to the Chinese adult obesity diagnostic criteria [15], the subjects were divided into two groups: 28 cases of normal-weight group (18.5kg/m^2^ ≤ BMI < 24kg/m^2^) and 33 cases of overweight/obesity group (BMI ≥ 24.0kg/m^2^). The study was approved by the ethics committee of Zhengzhou University.

### Inclusion criteria

(1) Adults to undergo abdominal surgery, such as appendicitis surgery, abdominal external hernia surgery, gallstone surgery; (2) The patient is mentally normal, without consciousness disorder, and can communicate fluently; (3) The patient voluntarily participates and signs an informed consent.

### Exclusion criteria

(1) Pregnant women, patients with disabilities or mental disorders, secondary obesity, malignant tumors; (2) Patients with infectious diseases such as hepatitis B, AIDS, and tuberculosis; (3) Patients taking adrenergic receptor blockers, antidepressants, or psychoactive drugs.

### Methods

### DNA extraction

During the operation, 1-2cm^2^ of abdominal SAT and OAT were taken out from the subjects, immediately washed with sterile saline and stored in liquid nitrogen.

### Methylation detection of CIDEB gene promoter

DNA extraction kit (TIANGEN, Beijing, China) was used to extract DNA, and the CIDEB promoter methylation level was detected by the MethylTarget method. The methylation detection was assisted by Genesky Biotechnologies Inc., Shanghai. Specific steps are as follows: (1) Agarose gel electrophoresis was used to detect the integrity of genomic DNA, and then Nanodrop 2000 (NanoDrop Technologies, Wilmington, DE, USA) was used to detect the quality of genomic DNA. (2) Design and optimization of primers. (3) Panel optimization of multiplex PCR primers: The primers optimized by step (2) were mixed into multiple PCR primers panel, and then the composition and concentration of primers in multiplex PCR panel were optimized by capillary electrophoresis. (4) Bisulfite treatment: Sample processing was performed using EZ DNA Methylation-Gold Kit (Zymo, Irvine, CA, USA) and convert cytosine C, which has not been methylated by genomic DNA, into uracil U. (5) Multiple PCR response of sample target fragment: The optimized multiplex PCR primer panel was used for multiplex PCR amplification with the transformed sample genome as the template. After quality control, the amplified products of all multiplex PCR primer panels with the same sample genomic DNA as template were mixed, and the amount of amplified products of each primer site was ensured to be equivalent. (6) Adding specific tag sequence to the sample: Using the primers with Index sequence, the specific tag sequence compatible with the Illumina platform (Illumina, San Diego, CA, USA) was introduced to the end of the library by PCR amplification. (7) High throughput sequencing: The Index PCR products of all samples were mixed equally to obtain the final MethylTarget sequencing library, whose fragment length distribution was verified by Agilent 2100 bioAnalyzer (Agilent Technologies, Santa Clara, CA, USA). After accurate quantification of the library molar concentration, the high-throughput sequencing was carried out on the Illumina Hiseq platform (Illumina, San Diego, CA, USA) with a 2×150bp double-terminal sequencing mode.

### Statistical analysis

The data were analyzed with IBM SPSS Statistics 23.0 (IBM Corp, Armonk, NY, USA). Basic characteristics of the subjects were described with mean and standard deviation $(\bar{x}\pm s)$ for quantitative variables or frequency (percentage) n (%) for qualitative variables. Two independent sample *t* test was used to compare the differences between normal-weight and overweight/obesity groups. Paired *t* test was used to test the methylation level of SAT and OAT. All *P*-values were 2-tailed, and the level of significance was set at *α* = 0.05.

### Availability of data and materials

The datasets generated and/or analyzed during the current study are available.

### Ethics approval

The study was approved by the ethics committee of Zhengzhou University.

## RESULTS

### The characteristics of subjects

According to the inclusion and exclusion criteria, 61 adult patients were included, of which both abdominal SAT and OAT were collected from 42 patients, and only one type of adipose tissue was collected from 19 patients. The average age of subjects was 51.87 years old. There were 28 cases in the normal-weight group, including 5 males and 23 females; 33 cases in the overweight/obesity group, including 5 males and 28 females. There was no statistical difference in gender distribution between two groups (*F063^2^*=0.081, *P*=0.776). The results of two independent sample *t* test showed that weight and BMI of overweight/obesity group were higher than normal-weight group (*P*<0.05), but there was no statistical difference in indicators such as age, height, waist-to-hip ratio, FPG, total cholesterol (TC), total triglyceride (TG), high density lipoprotein (HDL), low density lipoprotein (LDL) (*P*>0.05). Details were shown in Table 1.

**Table 1:** Comparison of general information between different BMI groups $(\bar{x}\pm s)$.

**Index**	**Normal-weight group**	**Overweight/obesity group**	***t***	***P***
	**(n=28)**	**(n=33)**		
Age	54.33±16.54	49.85±11.38	1.241	0.220
Height (cm)	160.65±6.34	161.65±6.00	-0.628	0.532
Weight (kg)	53.63±6.52	70.77±8.28	-7.225	<0.001
BMI (kg/m^2^)	21.91±1.42	27.05±2.50	-9.638	<0.001
WHR	0.90±0.05	0.92±0.07	-0.849	0.399
SBP (mmHg)	123.56±15.99	124.39±12.84	-0.225	0.823
DBP (mmHg)	74.22±9.56	74.27±7.60	-0.023	0.982
FPG (mmol/L)	5.30±1.31	6.45±4.13	-1.501	0.141
TC (mmol/L)	4.23±1.14	4.59±0.98	-1.324	0.191
TG (mmol/L)	1.59±1.36	2.24±1.64	-1.665	0.101
HDL (mmol/L)	1.06±0.31	1.10±0.26	-0.543	0.589
LDL (mmol/L)	2.55±1.25	2.27±0.94	1.004	0.320

BMI, body mass index; WHR, waist-hip ratio; SBP, systolic blood pressure; DBP, diastolic blood pressure; FPG, fasting plasma glucose; TC, total cholesterol; TG, total triglyceride; HDL, high density lipoprotein; LDL, low density lipoprotein.

### CIDEB gene promoter CpG methylation sites and the methylation level heatmap

**Figure 1:**
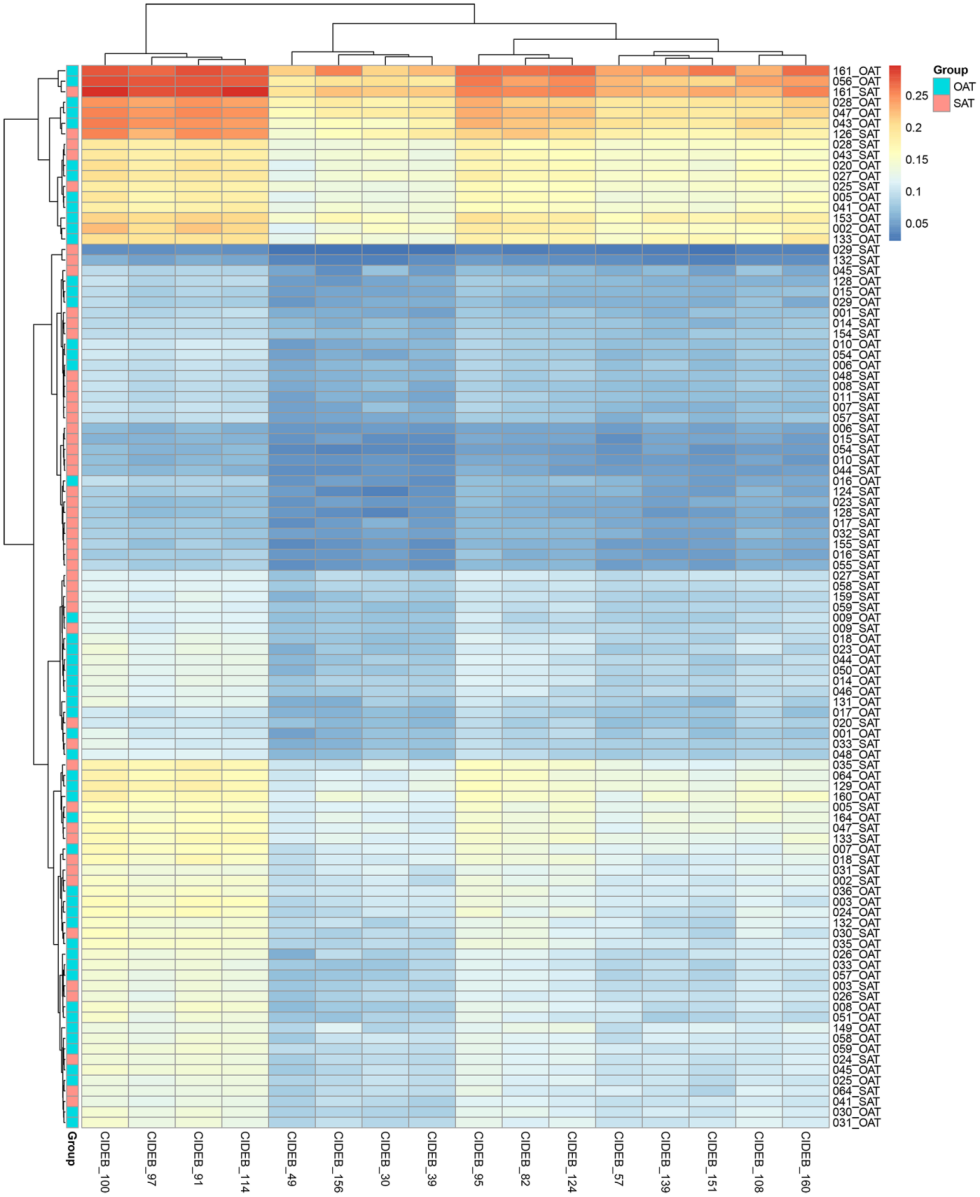
Methylation heatmap of CIDE-B gene promoter CpG sites.

In this study, 16 CpG methylation sites in CIDEB gene (Chr14: 24780614 to Chr14: 24780744) were detected. The various CpG sites and their corresponding physical locations in the chromosome were shown in Table 2. The methylation heatmap of CpG sites was shown in Figure 1.

**Table 2:** CpG sites of CIDEB gene promoter and their corresponding physical locations in the chromosome.

**CpG sites**	**Locations in chromosome**	**CpG sites**	**Locations in chromosome**
30	Chr14: 24780744	100	Chr14: 24780674
39	Chr14: 24780735	108	Chr14: 24780666
49	Chr14: 24780725	114	Chr14: 24780660
57	Chr14: 24780717	124	Chr14: 24780650
82	Chr14: 24780692	139	Chr14: 24780635
91	Chr14: 24780683	151	Chr14: 24780623
95	Chr14: 24780679	156	Chr14: 24780618
97	Chr14: 24780677	160	Chr14: 24780614

### Comparison of the methylation level of CIDEB gene promoter CpG sites between normal-weight and overweight/obesity groups

The results of two independent sample *t* test showed that there was no significant difference in CpG site methylation level between normal-weight and overweight/obesity groups (*P*>0.05). Details were shown in Table 3.

**Table 3:** Comparison of CIDEB gene promoter methylation level between different BMI groups $(\bar{x}\pm s)$.

**Adipose tissue**	**Methylation sites**	**Normal-weight group**			**Overweight/obesity group**		***P***
		**n**	** $(\bar{x}\pm s)$ **		**n**	** $(\bar{x}\pm s)$ **	
SAT	Chr14: 24780744	23	0.075±0.044		26	0.088±0.033	0.240
	Chr14: 24780735	23	0.071±0.043		26	0.083±0.035	0.314
	Chr14: 24780725	23	0.069±0.039		26	0.078±0.031	0.325
	Chr14: 24780717	23	0.082±0.045		26	0.093±0.035	0.332
	Chr14: 24780692	23	0.091±0.049		26	0.105±0.039	0.280
	Chr14: 24780683	23	0.110±0.053		26	0.124±0.044	0.337
	Chr14: 24780679	23	0.098±0.049		26	0.111±0.040	0.308
	Chr14: 24780677	23	0.106±0.052		26	0.117±0.042	0.405
	Chr14: 24780674	23	0.114±0.055		26	0.127±0.045	0.393
	Chr14: 24780666	23	0.087±0.042		26	0.099±0.036	0.314
	Chr14: 24780660	23	0.110±0.055		26	0.120±0.044	0.482
	Chr14: 24780650	23	0.091±0.049		26	0.104±0.039	0.310
	Chr14: 24780635	23	0.080±0.043		26	0.092±0.034	0.268
	Chr14: 24780623	23	0.077±0.045		26	0.093±0.034	0.155
	Chr14: 24780618	23	0.071±0.045		26	0.084±0.032	0.243
	Chr14: 24780614	23	0.085±0.049		26	0.098±0.035	0.305
OAT	Chr14: 24780744	25	0.096±0.037		27	0.109±0.039	0.247
	Chr14: 24780735	25	0.100±0.040		27	0.107±0.039	0.489
	Chr14: 24780725	25	0.087±0.038		27	0.096±0.037	0.426
	Chr14: 24780717	25	0.109±0.042		27	0.115±0.041	0.626
	Chr14: 24780692	25	0.126±0.046		27	0.134±0.044	0.516
	Chr14: 24780683	25	0.149±0.047		27	0.157±0.047	0.533
	Chr14: 24780679	25	0.133±0.046		27	0.142±0.046	0.473
	Chr14: 24780677	25	0.143±0.047		27	0.150±0.047	0.574
	Chr14: 24780674	25	0.155±0.046		27	0.161±0.048	0.680
	Chr14: 24780666	25	0.117±0.041		27	0.125±0.042	0.496
	Chr14: 24780660	25	0.145±0.046		27	0.154±0.047	0.461
	Chr14: 24780650	25	0.122±0.046		27	0.132±0.046	0.428
	Chr14: 24780635	25	0.109±0.044		27	0.119±0.041	0.430
	Chr14: 24780623	25	0.105±0.047		27	0.115±0.040	0.400
	Chr14: 24780618	25	0.097±0.046		27	0.108±0.041	0.360
	Chr14: 24780614	25	0.115±0.048		27	0.126±0.046	0.431

SAT, subcutaneous adipose tissue; OAT, omental adipose tissue.

### Comparison of the methylation level of CIDEB gene promoter between SAT and OAT

The results of paired *t* test showed that 16 CpG sites detected were statistically different between two groups, and the methylation level of these CpG sites in the OAT was higher than that in the SAT (Table 4).

**Table 4:** CIDEB gene methylation sites different statistically between SAT and OAT $(\bar{x}\pm s)$.

**Methylation sites**	**SAT**	**OAT**	***t***	***P***
Chr14: 24780744	0.083±0.039	0.100±0.038	4.385	<0.001
Chr14: 24780735	0.079±0.037	0.102±0.041	5.564	<0.001
Chr14: 24780725	0.076±0.035	0.089±0.038	3.878	<0.001
Chr14: 24780717	0.089±0.040	0.110±0.041	4.836	<0.001
Chr14: 24780692	0.099±0.043	0.127±0.046	5.994	<0.001
Chr14: 24780683	0.119±0.047	0.151±0.047	6.423	<0.001
Chr14: 24780679	0.107±0.044	0.135±0.046	6.484	<0.001
Chr14: 24780677	0.114±0.046	0.144±0.046	6.299	<0.001
Chr14: 24780674	0.122±0.049	0.155±0.047	6.398	<0.001
Chr14: 24780666	0.095±0.038	0.118±0.040	5.794	<0.001
Chr14: 24780660	0.117±0.048	0.147±0.047	5.928	<0.001
Chr14: 24780650	0.099±0.044	0.124±0.046	5.882	<0.001
Chr14: 24780635	0.088±0.038	0.112±0.042	6.149	<0.001
Chr14: 24780623	0.087±0.040	0.108±0.044	5.207	<0.001
Chr14: 24780618	0.080±0.039	0.100±0.044	5.147	<0.001
Chr14: 24780614	0.094±0.042	0.118±0.047	5.772	<0.001

SAT, subcutaneous adipose tissue; OAT, omental adipose tissue.

### Comparison of CIDEB gene methylation haplotypes between normal-weight group and overweight/obesity group

Table 5 showed that there were 3 haplotypes with statistical differences between normal-weight and overweight/obesity groups, including 2 in SAT and 1 in OAT.

**Table 5:** Haplotypes of CIDEB gene promoter methylation different statistically between different BMI groups.

**Adipose tissue**	**Haplotype**	**Normal-weight group (n = 23)**	**Overweight/obesity group (n = 26)**	***P***
SAT	ttttttttttcttttt	0.010±0.003	0.008±0.002	0.011
	ttttttttttttttct	0.006±0.002	0.005±0.001	0.016
OAT^a^	ttttttttcttttttt	0.010±0.002	0.008±0.002	0.027

SAT, subcutaneous adipose tissue; OAT, omental adipose tissue.

^a^OAT samples in normal-weight group and overweight/obesity group are 25,27 respectively.

## DISCUSSION

In recent years, the role of epigenetics has attracted more and more attention in the process of studying the pathogenesis of diseases. DNA methylation is a well-studied epigenetic modification, which only affects the transcription activity of genes and does not change the DNA sequence. A genome-wide analysis found that methylation levels of some genes were associated with different somatotypes and body composition of preschool children [16]. The previous study of our group showed that methylation level of PRDM16 gene promoter was higher in overweight/obese people [17]. In a large-scale study investigating DNA methylation in CD4+ T-cells [18], 8 CpG sites were associated with BMI and five with WC. Therefore, DNA methylation can regulate the growth and development of adipose tissue, which plays an important role in the occurrence and development of metabolic diseases related to obesity [19, 20].

There are three members of the CIDE protein family, including CIDEA, CIDEB, and CIDEC/fat-specific protein 27 (FSP27, in rodent), which were originally thought to be involved in apoptosis in mammals [21]. Along with the deepening of the study, it has been discovered that CIDE proteins are key factors in controlling multiple lipid metabolism pathways and maintaining lipid homeostasis [22], and are closely related to the occurrence and development of obesity, diabetes, and fatty liver [9, 23, 24]. Whether in brown or white adipocytes, the lack of CIDE family proteins can cause the lipid droplets in these cells to decrease in size, increase in number, and accelerate fat degradation [22, 25].

Our study found that methylation value of each CpG site of CIDEB gene in overweight/obesity group was higher than that of normal-weight group, but there was no statistical difference. Possible reasons are as follows: (1) Due to human subjects, it is difficult to obtain adipose tissue, resulting in a small sample size; (2) Obesity is a complex disease, and its occurrence is the result of the interaction of multiple genes [26, 27], so methylation status of a single gene promoter sequence may not be significant. Therefore, the combined study of multiple gene promoter methylation may be more helpful to explain the molecular mechanism of the occurrence and development of obesity, and DNA methylation can be explored at the genome-wide level in future studies.

This is the first study of the CIDEB gene methylation level in adipose tissue. We found that methylation levels of the CIDEB promoter in OAT were higher than that in SAT, indicating that expression of CIDEB in SAT was more than that of OAT, which was consistent with the fact that subcutaneous fat storage is higher than that of OAT. According to functional and anatomical differences, WAT is divided into SAT and OAT [28]. SAT is the largest fat storage tissue. When the energy storage of SAT is saturated, fat is ectopically stored in viscera [29]. Therefore, higher level of CIDEB expression in the SAT made white fat accumulate preferentially in the subcutaneous layer.

In terms of genetics, chronic non-communicable diseases are caused by the interaction of multiple genes or mutations within genes, rather than the isolated effect of a single polymorphic site. Haplotype refers to the combination of alleles at multiple sites that are inherited together on the same chromosome. Haplotype analysis is an accurate and reliable method for discovering the association between genome structure and diseases [30]. We found that 3 haplotypes were related to overweight or obesity, which indicated that regulation of CIDEB expression is not a single-site mutation, but a combination of multiple-site mutations.

This study is the first to report the association of the CIDEB gene promoter methylation level with overweight or obesity in abdominal adipose tissue of adults, which is a significant exploration to elucidate the epigenetic mechanism of obesity development. Moreover, the MethylTarget technology we used can accurately calculate the methylation level of each CpG site, which has the advantages of high accuracy, strong flexibility, and high-cost performance.

The relatively small sample size was a limitation of this study. Following the principle of informed consent, human tissue sampling is inconvenient, and it is indeed difficult to obtain both SAT and OAT. Moreover, due to the lack of relevant studies on methylation of CIDEB gene at present, the sample content cannot be determined by literature method. Through literature review, we found that the sample size of DNA methylation studies involving human tissues was generally small, while the sample size of studies obtaining both types of adipose tissue was even smaller. For example, a Spanish study [31] collected visceral adipose tissues (VAT) from 57 patients with colorectal cancer and 50 healthy controls to explore vitamin D receptor (VDR) expression and methylation in colorectal cancer. Helene A. Fachim et al. [32] performed SAT biopsy in 20 patients with impaired glucose regulation to evaluate the effect of lifestyle intervention on Caveolin-1 gene methylation and provided insights for targeted treatment of diabetes. To investigate the relationship between obesity and insulin resistance, Aneta Cierzniak et al. [33] collected VAT and SAT from 45 patients undergoing abdominal surgery. Tarryn Willmer et al. [34] obtained abdominal SAT and gluteal SAT from 27 obese and 27 normal weight urban-dwelling South African women and found that the methylation level of FK506-binding protein 51 kDa (FKBP5) gene was associated with obesity and insulin resistance. In conclusion, the sample size of this study is moderate, and the results of this study have a certain reference value, which can provide a theoretical basis for finding targets for the treatment of obesity.

## CONCLUSIONS

The methylation level of CIDEB gene promoter in abdominal SAT and OAT may be related to overweight or obesity in adults, and the specific regulatory mechanism needs to be further studied.
